# Adaptive Time–Frequency Segment Optimization for Motor Imagery Classification

**DOI:** 10.3390/s24051678

**Published:** 2024-03-05

**Authors:** Junjie Huang, Guorui Li, Qian Zhang, Qingmin Yu, Ting Li

**Affiliations:** 1 China Academy of Information and Communications Technology, Beijing 100191, China; 372657533@hrbeu.edu.cn (J.H.); zhangqian@caict.ac.cn (Q.Z.); 2 Key Laboratory of Internet and Industrial Integration Innovation, Beijing 100191, China; 3 Institute of Biomedical Engineering, Chinese Academy of Medical Sciences & Peking Union Medical College, Tianjin 300192, China; liguorui2021@student.pumc.edu.cn

**Keywords:** motor imagery, brain–computer interface, time–frequency segments, sparrow search algorithm

## Abstract

Motor imagery (MI)-based brain–computer interface (BCI) has emerged as a crucial method for rehabilitating stroke patients. However, the variability in the time–frequency distribution of MI-electroencephalography (EEG) among individuals limits the generalizability of algorithms that rely on non-customized time–frequency segments. In this study, we propose a novel method for optimizing time–frequency segments of MI-EEG using the sparrow search algorithm (SSA). Additionally, we apply a correlation-based channel selection (CCS) method that considers the correlation coefficient of features between each pair of EEG channels. Subsequently, we utilize a regularized common spatial pattern method to extract effective features. Finally, a support vector machine is employed for signal classification. The results on three BCI datasets confirmed that our algorithm achieved better accuracy (99.11% vs. 94.00% for BCI Competition III Dataset IIIa, 87.70% vs. 81.10% for Chinese Academy of Medical Sciences dataset, and 87.94% vs. 81.97% for BCI Competition IV Dataset 1) compared to algorithms with non-customized time–frequency segments. Our proposed algorithm enables adaptive optimization of EEG time–frequency segments, which is crucial for the development of clinically effective motor rehabilitation.

## 1. Introduction

Brain–computer interface (BCI) technology establishes communication between brains and computers or other devices without relying on peripheral nerves or muscles [1]. Among various types of signals from the brain, EEG is considered the most promising brain signal due to its portable, cost-effective, and non-invasive advantages. Motor imagery (MI) is one typical electroencephalography (EEG)-based paradigm for BCI development; the voltage fluctuation in the motor area cortex due to imaging a real movement or executing MI is similar to a large degree [2]. Specifically, it has been observed that imagination and finishing of a movement results in a power decrease and increase in EEG signals in the alpha (8~12 Hz) and beta (14~30 Hz) frequency bands. This phenomenon is recognized as event-related desynchronization (ERD) and event-related synchronization (ERS), and serves as the foundation for MI-BCI classification [3].

Spatial filtering and channel selection are commonly utilized in order to enhance the performance of MI-BCI. The common spatial pattern (CSP) algorithm and its related algorithms [4,5,6,7] serve to enhance the discernibility of MI-BCI mental states by maximizing the variance of one class while minimizing the variance of the other class. Jin et al. proposed a channel selection method based on brain network parameters using the Pearson coefficient to quantify the statistical relationship and select the optimal channels [5]. Zhang et al. incorporated an auto channel selection layer into the neural network, allocating weights based on the EEG channels’ contributions to MI signal classification, thereby facilitating adaptive channel selection [8].

The aforementioned algorithms have demonstrated some degree of enhancement in the performance of the MI-BCI systems. However, their effectiveness heavily relies on pre-adopted time truncation and frequency filtering. Most of the existing literature has set a broad frequency band (typically 8~30 Hz) and a non-customized time segment (typically a short period of time after onset of the MI cue). Nevertheless, the time–frequency characteristics of ERD/ERS evoked by different subjects vary. Therefore, a non-customized time–frequency segment leads to subpar classification results across different subjects. The appropriate time–frequency segments are very subject-dependent, which will be the main concern of this paper.

To address the challenge of selecting appropriate time–frequency segments, Kai et al. [9,10] proposed a band selection algorithm, namely, FBCSP, which decomposes signals into several frequency bands. Subsequently, CSP is applied to each band before combining and selecting the CSP features. Sliding windows and overlapping time–frequency segments are employed in other studies to further increase the optional time–frequency segments [11].

The aforementioned methods achieve the selection of time–frequency segments. However, they are hindered by limited optional time–frequency segments, which are dependent on the preset parameters. To address this issue, we proposes the sparrow search algorithm (SSA) correlation-based channel selection (CCS)-regularized common spatial pattern (RCSP) algorithm, which adaptively explores the optimal time–frequency segments and selects appropriate channels to enhance the performance of feature extraction for subjects. Initially, the SSA is employed to optimize the time–frequency segments. Subsequently, the CCS algorithm is utilized to identify EEG channels exhibiting significant correlation. Following this, the RCSP algorithm is implemented to extract signal features from the aforementioned selected channels. Lastly, the support vector machine (SVM) is employed to classify the feature vectors.

Experiments were conducted to verify the effectiveness and generalization of the algorithm across varying subjects and channels, utilizing the publicly available BCI Competition III Dataset IIIa, autonomously collected data from the Chinese Academy of Medical Sciences, and the BCI Competition IV Dataset 1, respectively. The results showed that the recognition efficiency of the proposed algorithm outperformed the algorithms in the comparison group, demonstrating its suitability for diverse subjects and channels. The proposed algorithm is expected to promote the application of MI-BCI in the field of motor rehabilitation by customizing personalized time–frequency segments for subjects.

The major contributions of this study are listed as follows:

- A new approach to optimize the time–frequency segment is defined, aiming to provide a subject-specific time–frequency segment.
- We develop a novel framework for MI decoding. Rather than employing a non-customized time–frequency segment, our approach adaptively explores the optimal time–frequency segment without constraints on limited optional segments.
- To verify the effectiveness of the proposed algorithm, three MI-EEG datasets are included for classification experiments, and the proposed algorithm shows competitive and robust performance.

The rest of the paper is organized as follows: In Section 2, some related work is reviewed at first. Section 3 introduces the details of the proposed methods, including optimization of time–frequency segments, channel selection, feature extraction, and classification. Section 4 elaborates upon data preprocessing, the overall framework of the algorithm, and the results of the experiment. Section 5 analyzes the experimental results and discusses the proposed algorithm. Section 6 concludes the study and points out future work.

## 2. Related Works

The feature extraction and channel selection process is key to the MI decoding process. Feature extraction in MI decoding faces the challenge of a low signal-to-noise ratio, inherent non-stationary nature, and high inter-subject variability. Currently, many MI feature extraction algorithms have been developed to enhance the performance of MI-BCI [12,13,14]. CSP is a powerful spatial filtering feature extraction method that provides a filter to maximize the variance of one class of signals while minimizing the variance from the other class. Many variations of CSP have been proposed to enhance its performance, among which the regularized CSP exhibits promising results [5,6,7]. However, the effectiveness of CSP heavily relies on pre-adopted time truncation and frequency filtering. Moreover, the high inter-subject variability means that the optimal time–frequency segments for extracting features could be different for different subjects. Hence, a broad frequency range (typically 8~30 Hz) and a non-customized time segment are commonly used [10]. Exhaustive search is another effective method for addressing this issue; however, the impractical computational time requirements hinder its practical application.

To achieve automatic selection of frequency bands, Ang et al. proposed the Filter Bank Common Spatial Pattern (FBCSP) method, which decomposes the signal into multiple frequency bands through bandpass filtering. Subsequently, CSP is applied to each band, followed by the use of a feature selection algorithm to automatically select frequency bands and their corresponding CSP features [10]. However, there are still some limitations involved. First, there is a lack of attention to the optimal time segment selection. Time segment selection is another important issue in the decoding of MI EEG signals, aiming to cover the interval strongly correlated with motor intention and remove unrelated sampling points. Second, the search space for time–frequency segments is dependent on the choice of initial parameters, and the limitations of the available time–frequency segments make it difficult to find subject-specific optimal segments.

To address the first concern, Peterson et al. decomposed the EEG signals into multiple time windows. In each window, they extracted FBCSP features, resulting in penalized time–frequency band CSP features [15]. They simultaneously considered the selection of time segments and frequency bands and achieved a more stable classification accuracy compared to FBCSP. To address the second concern, a temporally constrained sparse group spatial pattern applies bandpass filtering to the raw EEG data across a set of overlapping filter bands [16]. This method enhances the variety of selectable time–frequency segments. The utilization of sliding windows and overlapping time–frequency segments serves to augment the pool of available combinations; nonetheless, the selection of time–frequency segments remains contingent upon initial parameters, presenting a challenge in terms of the freedom of time–frequency segment selection. Hence, achieving time–frequency band optimization without being limited by selection space is a problem that needs to be solved.

The main types of channel selection algorithms encompass those employing neural networks and those utilizing functional connectivity methods. Channel selection algorithms utilizing functional connectivity methods are based on the theory that signals from the same type of motor imagery task are more similar, while signals from different types of motor imagery tasks exhibit more significant differences. By sorting the correlation across different channels, the optimal channels are selected. In [17], cross-correlation is proposed to evaluate the correlation across channels using the Pearson coefficient [18].

Channel selection algorithms employing neural networks have gained attention in recent years. In [19], researchers considered EEG channels as a group of neural networks and studied EEG channel selection. In [20], a convolutional neural network with specific channels was employed as a feature extractor to enhance the separability of classifications. Subsequently, a collection of deep sparse autoencoders was utilized to select the optimal channels. Sun [21,22,23,24] et al. proposed a graph-based channel selection method, which automatically selected a specified number of optimal channels in the graph convolutional neural network by utilizing the time-domain correlation of signals. 

Optimization algorithms have also been applied to enhance channel selection. In [25], a neural evolutionary channel selection algorithm was proposed based on an improved particle swarm optimization algorithm for channel selection, and CSP was used for feature extraction.

In this study, we propose an adaptive time–frequency segment optimization algorithm to overcome the selection space limitation and customize personalized time–frequency segments for subjects. Furthermore, we incorporate CCS for channel selection and utilize RCSP for feature extraction to enhance the decoding performance of MI.

## 3. Materials and Methods

### 3.1. Datasets

The performance of the proposed method was validated on three datasets: the BCI Competition III Dataset IIIa, the Chinese Academy of Medical Sciences autonomous dataset, and the BCI Competition IV Dataset 1.

#### 3.1.1. BCI Competition III Dataset IIIa (DS1)

**Figure 1:**
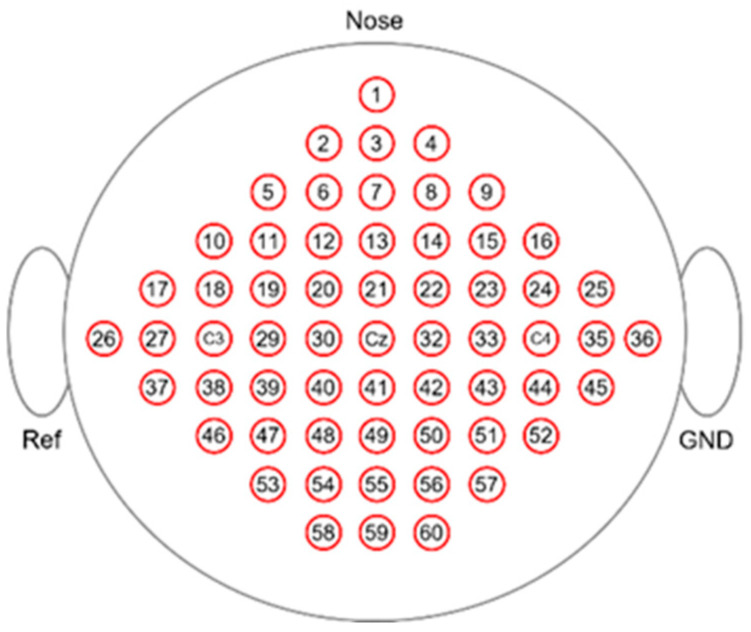
Distribution of channels of DS1. The circles and different numbers correspond to the positions of EEG electrodes.

The BCI Competition III Dataset IIIa is a four-class EEG dataset (left hand, right hand, foot, tongue; the first two actions were used for the experiments in this paper) which was acquired using a 64-channel Neuroscan EEG amplifier, with the left mastoid for reference and the right mastoid as ground. The sampling frequency for the data was set at 250 Hz, and the recording was conducted using 60 electrodes [26,27]. The distribution of the channels is shown in Figure 1.

**Figure 2:**
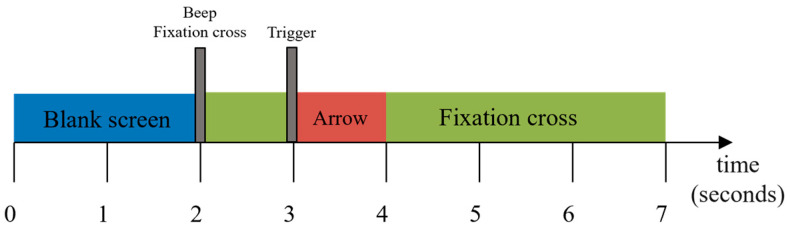
Timeline of one trial of DS1.

During the experimental sessions, the subjects sat in chairs with armrests and performed different MI tasks according to cues, and the order of cues was random. Each trial commenced with a period of silence lasting 2 s, then an acoustic stimulus and a cross on the screen indicated the start of the trial. From 3 s to 7 s, the subjects were asked to performed the indicated mental task. The timeline is shown in Figure 2.

The dataset was recorded from three subjects (K3b, K6b, and L1b), and the numbers of trials per class were 90 and 60 for participants K3b and L1b, respectively. Due to the considerable variability in the accuracy of the K6b data reported in different studies, as well as suspicions regarding potential errors in the true labels, the credibility of this particular dataset was not deemed sufficiently high. Consequently, only the K3b and L1b datasets were employed as experimental data for the purposes of this paper.

#### 3.1.2. Chinese Academy of Medical Sciences Autonomous Dataset (DS2)

**Figure 3:**
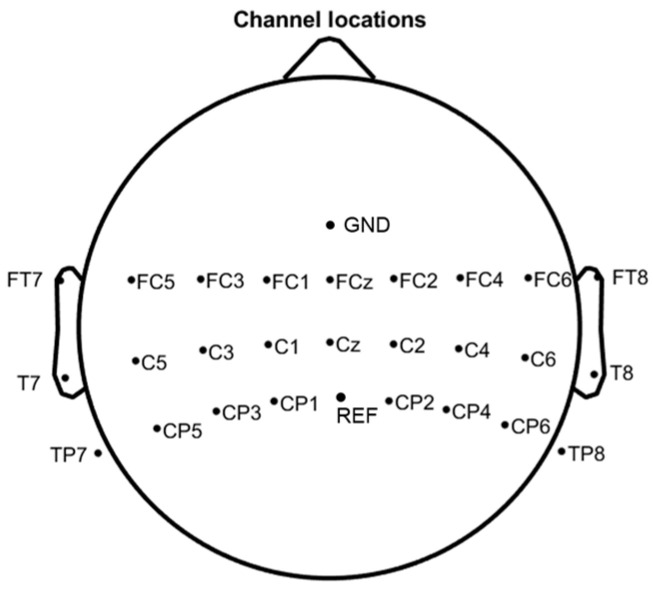
Distribution of channels of DS2.

The Chinese Academy of Medical Sciences autonomous dataset is a two-class EEG dataset which was acquired using a 64-channel Neuracle EEG amplifier, with the REF channel for reference and the GND channel as ground. The sampling frequency for the data was set at 1000 Hz. The electrode locations adhered to the international 10–20 system. The distribution of the channels is shown in Figure 3.

**Figure 4:**
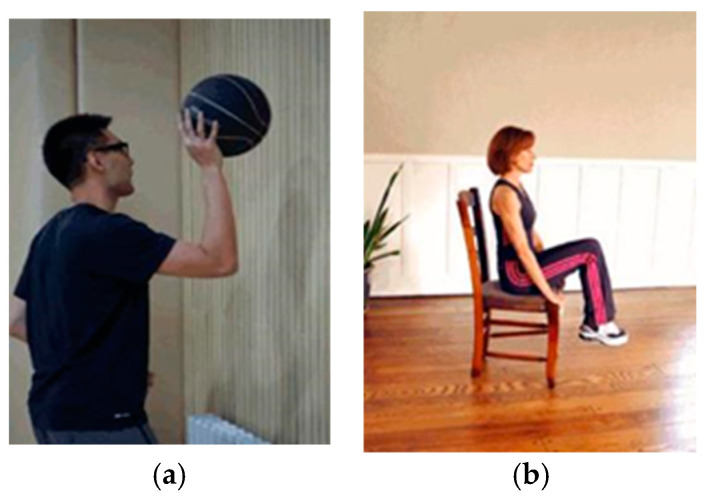
Motor imagery tasks of DS2. (**a**) Right hand ball bouncing; (**b**) foot lifting.

**Figure 5:**
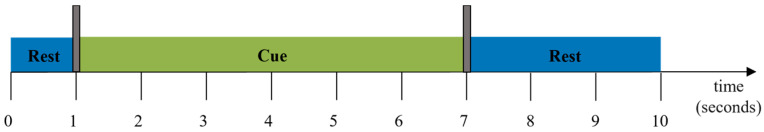
Timeline of one trial of DS2.

Subjects performed 2 MI tasks of right-handed ball bouncing and foot lifting; the movements are shown in Figure 4. In each trial, the experiment commenced with a rest period of 1 s. Then, a visual stimulus in the form of a movement picture was presented to prompt the subjects to mentally simulate the depicted movement. Following a duration of 6 s for MI, the subjects were alerted to transition into a 3 s period of rest through the activation of a system buzzer and the display of a black screen. Each subject participated in 320 trials, with an equal distribution of 160 trials for each type of movement. The timeline is shown in Figure 5.

The recording process involved nine subjects (A01~A09) in DS2 from the Chinese Academy of Medical Sciences, Tiangong University, and Tianjin Medical University. All subjects were right-handed, with an average age of 22.3 +/− 2.9 years. All subjects provided their informed consent (Supplementary Materials S1).

#### 3.1.3. BCI Competition IV Dataset 1 (DS3)

**Figure 6:**
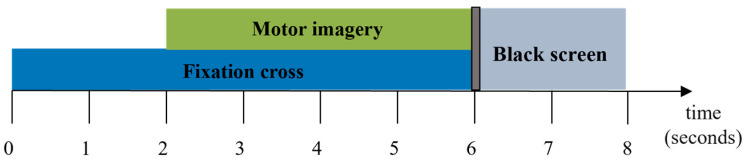
Timeline of one trial of DS3.

The BCI Competition IV Dataset 1 was acquired from 59 EEG channels [28,29]. For each subject, two classes of motor imagery were chosen from the three classes: left hand, right hand, and foot. Each trial began with a 2 s display of a fixation cross at the center of the screen, followed by the presentation of cues (arrows pointing left, right, or down) for 4 s. Participants were instructed to perform the corresponding MI task during this period. Subsequently, a 2 s blank screen ensued. The fixation cross overlaid the cues and was displayed for 6 s. Each of the two runs consisted of 100 trials, and the timeline for each trial is depicted in Figure 6. 

DS3 comprises recordings from 7 participants engaged in MI tasks without feedback. Data from participants ‘a’, ‘b’, ‘g’, ‘f’ were utilized in this study, and ‘c’, ‘d’, and ‘e’ were excluded due to the fact that they were artificially generated.

### 3.2. Method

#### 3.2.1. Sparrow Search Algorithm-Based Time–Frequency Segment Optimization

SSA is a swarm intelligence optimization algorithm that is mainly inspired by the foraging and antipredator behavior of sparrows. Since the optimal time–frequency segments of EEG vary from subject to subject [30], non-customized time–frequency segments cannot represent the optimal segments for different individuals. To solve this problem, the SSA-based algorithm is utilized to obtain the optimal time–frequency segments of different individual EEG signals. During the search process, sparrows are classified into 3 roles [31]:

(1) Producer, responsible for providing appropriate exploration areas or directions for all sparrows seeking to optimize the time–frequency segments of EEG signals;
(2) Scrounger, following the sparrows that can indicate the direction of better time–frequency segments;
(3) Scout, aware of potential shortcomings in the current process of optimizing the time–frequency segment range of EEG signals, and seeking better time–frequency segments by escaping from the current position.

The sparrow position parameters can be represented by the following matrix:

(1) $$X=\left(\begin{array}{cc} \begin{array}{cc} X_{1,1} & X_{1,2}\\ X_{2,1} & X_{2,2} \end{array} & \begin{array}{cc} \cdots & X_{1,d}\\ \cdots & X_{2,d} \end{array}\\ \begin{array}{cc} \vdots & \vdots \\ X_{n,1} & X_{n,2} \end{array} & \begin{array}{cc} \vdots & \vdots \\ \cdots & X_{n,d} \end{array} \end{array}\right),$$

where n is the number of sparrows and d denotes the parameter dimension, and the value of each row $X_{i}$ represents the time–frequency segment corresponding to the ith sparrow, i.e., the frequency start point $f_{start}\;$($X_{i,1}$), frequency width $f_{width}\;$($X_{i,2}$), time start point $t_{start}$ ($X_{i,3}$), and time width $t_{width}(X_{i,4})$. 

Sparrow roles are classified according to the fitness value, which is derived from the classifier’s accuracy. It can be expressed by the following vector:

(2) $$F=\left(\begin{array}{c} f\left(\left[\begin{array}{cc} \begin{array}{cc} X_{1,1} & X_{1,2} \end{array} & \begin{array}{cc} \cdots & X_{1,d} \end{array} \end{array}\right]\right)\\ f\left(\left[\begin{array}{cc} \begin{array}{cc} X_{2,1} & X_{2,2} \end{array} & \begin{array}{cc} \cdots & X_{2,d} \end{array} \end{array}\right]\right)\\ \begin{array}{c} \vdots \\ f\left(\left[\begin{array}{cc} \begin{array}{cc} X_{n,1} & X_{n,2} \end{array} & \begin{array}{cc} \cdots & X_{n,d} \end{array} \end{array}\right]\right) \end{array} \end{array}\right),$$

$f\left(X_{i}\right)$ denotes the fitness value obtained based on $X_{i}$. The position of the sparrow with the lowest fitness value corresponds to the current optimal time–frequency segment. The k sparrows with lower fitness are classified as producers. Since producers are responsible for guiding other sparrows, they search in a broad range around themselves for better time–frequency segments. The producer location is updated as below:

(3) $$X_{i,j}^{t+1}=\left\{\begin{array}{ll} X_{i,j}^{t}\cdot \exp\left(\frac{-i}{\alpha \cdot {iter}_{max}}\right), & R_{2}<\mathrm{ST}\\ X_{i,j}^{t}+Q\cdot L, & R_{2}\geq \mathrm{ST} \end{array}\right.,$$

where t denotes the current iteration, j=1,2,…,d. ${iter}_{max}$ is the largest number of iterations, $\alpha \in \left(0\right.,\left.1\right]$ is a random number, Q is a random number obeying normal distribution, and $X_{i,j}^{t}$ denotes the jth dimensional parameter of the ith sparrow in t iteration. L is a 1×d matrix with all elements equal to 1. $R_{2}\in \left[0\right.,\left.1\right]$ is alarm value and $ST\in \left[0.5\right.,\left.1\right)$ is the safety threshold; both of them are random numbers. When $R_{2}\geq ST$, producers will fly to other areas, searching for better time–frequency segments. On the other hand, when $R_{2}<ST$, it means that no predator is around and the producers enter extensive search mode. This mechanism prevents the algorithm from falling into local optima.

The scrounger position is updated as follows:

(4) $$X_{i,j}^{t+1}=\left\{\begin{array}{l} Q\cdot \exp\left(\frac{X_{worst}^{t}-X_{i,j}^{t}}{i^{2}}\right),\;\;\;\;\;\;\;\;\;\;\;\;\;\;\;\;\;\;\;\;\;\;\;i>\frac{n_{2}}{2}\\ X_{best}^{t}+\\ \frac{1}{d}\sum_{j=1}^{d}\left(rand\left\{-1,1\right\}\cdot \left(\left|X_{i,j}^{t}{-X}_{best}^{t}\right|\right)\right),\;i\leq \frac{n}{2} \end{array}\right.,$$

where $X_{worst}^{t}$ and $X_{best}^{t}$ are the global worst and best positions in t iterations, respectively. $n_{2}$ denotes the numbers of scroungers; if $i>\frac{n_{2}}{2}$, the current time–frequency segment is not suitable for the subject under investigation and the scrounger will leave the current position. If $i\leq \frac{n_{2}}{2}$, the scrounger will approach the producer corresponding to the optimal time–frequency segment.

Some of the sparrows are aware of the danger; these sparrows are scouts, and they will abandon their current time–frequency segments and move to new locations. The scout is updated as below:

(5) $$X_{i,j}^{t+1}=\left\{\begin{array}{c} X_{best}^{t}+\beta \cdot \left(X_{i,j}^{t}-X_{best}^{t}\right),\;\;f_{i}\neq f_{g}\\ X_{i,d}^{t}+K\cdot \left(\;\frac{X_{i,j}^{t}-X_{worst}^{t}}{\left|f_{i}-f_{w}\right|+\varepsilon }\right),\;\;f_{i}=f_{g} \end{array}\right.,$$

where β and K are random numbers,β conforms to the standard normal distribution, and $K\in \left(0\right.,\left.1\right]$ denotes the direction of the sparrow. ε is a small constant to avoid zero-division errors. Here, $f_{i}$ is the fitness value of the ith sparrow. $f_{g}$ and $f_{w}$ denote the best and worst fitness values, respectively. If $f_{i}=f_{g}$, the sparrow will need to move closer to others; otherwise, it will move to the vicinity of the current optimal position.

After comparing using grid search method to determine the ratio, producers and scroungers are sorted by fitness, constituting 70% and 30% of the total, respectively, while scouts are randomly generated in the population with a ratio of 0.2.

#### 3.2.2. Correlation-Based Channel Selection Algorithm

The channels associated with MI should contain common information for performing the same task [5]. Therefore, removing less correlated channels can improve feature extraction. By calculating the Pearson correlation coefficients between signals from different channels, the CCS algorithm can select the subject-specific best channels that are associated with MI [32]. Two steps need to be taken in the CCS algorithm:

First, data normalization: z-score normalization is applied to the EEG signals with the assistance of the sklearn package in Python, so that the mean of EEG data is 0 and the standard deviation is 1. Before normalization, the signal undergoes time segmentation and frequency filtering, but without artifact removal.

Second, based on the Pearson correlation coefficient, the statistical relationship between every pair of channels is quantified. The Pearson correlation coefficient is calculated as below:

(6) $$\rho \left(X,Y\right)=\frac{1}{n-1}\sum_{i=1}^{n}\left(\frac{X_{i}-\bar{X}}{\sigma_{X}}\right)\left(\frac{Y_{i}-\bar{Y}}{\sigma_{Y}}\right),$$

where X and Y are the observed variables, $\bar{X}$ and $\bar{Y}$ are the mean variables, n is the sample size, and $\sigma_{X}$ and $\sigma_{Y}$ are the standard deviations of the variables. The matrix of correlation coefficients of the N channels is defined as:

(7) $$R=\left(\begin{array}{cc} \begin{array}{cc} \rho \left(X_{1},X_{1}\right) & \rho \left(X_{1},X_{2}\right)\\ \rho \left(X_{2},X_{1}\right) & \rho \left(X_{2},X_{2}\right) \end{array} & \begin{array}{cc} \cdots & \rho \left(X_{1},X_{N}\right)\\ \cdots & \rho \left(X_{2},X_{N}\right) \end{array}\\ \begin{array}{cc} \vdots & \vdots \\ \rho \left(X_{N},X_{1}\right) & \rho \left(X_{N},X_{2}\right) \end{array} & \begin{array}{cc} \vdots & \vdots \\ \cdots & \rho \left(X_{N},X_{N}\right) \end{array} \end{array}\right),$$

where N is the total number of channels. The mean of the ith row represents the correlation between the ith channel and other channels, while a higher correlation indicates that channel i is more related to other channels. Therefore, $N_{t}\times N_{s}$ highly correlated channels are selected after $N_{t}$ trials, and the $N_{s}$ channels that most frequently appear in $N_{t}\times N_{s}$ recordings are chosen as the ideal channels.

#### 3.2.3. Feature Extraction Based on Regularized Common Spatial Pattern

The RCSP algorithm is an improved version of the CSP algorithm [5,7]. The CSP algorithm uses a spatial filter to maximize the variance for one class and minimize the variance for another class. The RCSP algorithm introduces regularization parameters α and β into the covariance calculation to obtain the regularized mean covariance matrix $Q^{class}\left(\alpha ,\beta \right)$, which is calculated as:

(8) $$Q^{class}\left(\alpha ,\beta \right)=\left(1-\beta \right)P^{class}+\frac{\beta }{N_{s}}trace\left(P^{class}\right)I,$$

where $trace\left(\cdot \right)$ represents the matrix trace, I represents the identity matrix, and $P^{class}$ is the average covariance matrix, which is calculated as follows:

(9) $$P^{class}\left(\alpha ,\beta \right)=\frac{\left(1-\alpha \right)\sum_{i=1}^{N_{tr}}C_{i}^{class}+\alpha \sum_{i=1}^{N_{tr}}{\hat{C}}_{i}^{class}}{N_{tr}},$$

where $C_{i}^{class}$ is the normalized covariance matrix and ${\hat{C}}_{i}^{class}$ is the pair-wise covariance, which are defined as:

(10) $$C_{i}^{class}=\frac{E_{i}E_{i}^{T}}{trace\left(E_{i}E_{i}^{T}\right)},$$

(11) $${\hat{C}}_{i}^{class}=cov\left(E_{i}^{T}\right),$$

where cov(·) is the function that calculates the pair-wise covariance of each channel. To maximize the variance of one class while minimizing the variance of the other class, a filter ω is defined as:

(12) $$\omega =argmin\frac{\omega^{T}Q^{c1}\omega }{\omega^{T}Q^{c2}\omega },$$

The above equation is transformed into the generalized eigenvalue problem by means of the Lagrange multiplier method:

(13) $$Q^{c1}\omega =\lambda Q^{c2}\omega ,$$

The eigenvectors of the m maximum and minimum eigenvalues are obtained as the optimal spatial filter $\omega_{2m}\in R^{N_{s}\times 2m}$, and the EEG signal features are extracted with the following equations:

(14) $$f=\ln\left(var\left(\omega_{2m}^{T}E\right)\right)$$

For the convenience of comparing the performance of the proposed algorithm on different datasets, the parameters m, α, and β are fixed at 2, 0.4, and 0.01, respectively.

#### 3.2.4. Feature Classification Based on Support Vector Machine

SVM is a binary classification model which is suitable for nonlinear and high-dimensional classification [33]. In this paper, a radial basis function (RBF) kernel function is used to transform the inner product in the high-dimensional feature space into the computed result of the kernel function in the low-dimensional feature space to reduce the computation’s complexity.

The penalty parameter C and the kernel function parameter g have a large impact on the SVM classification effect; thus, in the proposed method, we used a grid search method(C∈[0.001,0.01,0.1,1,10,100,1000] and g∈[0.001,0.01,0.1,1,10,100,1000]) to find their optimal values.

Generalizability is an important indicator of the practical application capability of the model. To improve generalizability, in addition to tuning by hyperparameters, the calculation method of model evaluation can be adjusted. In this paper, a five-fold cross-validation method was employed to estimate the accuracy of SVM, and the Sklearn package in Python was used.

## 4. Results

This section describes the experimental data processing framework, results, and performance of the proposed model and four benchmark models used for comparison.

### 4.1. Data Preprocessing

In this study, we underwent preprocessing using the EEGLAB toolbox [34] in Matlab (The MathWorks Inc., Natick, MA, USA) and the mne package in Python 3.6 (The Python Software Foundation, Wilmington, DE, USA). The preprocessing process included downsampling, filtering, time slicing, baseline correction, and artifact rejection.

For the DS1, the signals was downsampled to 250 Hz by the provider of the DS1. Since significant power changes occurred in the alpha (8~12 Hz) and beta (14~30 Hz) rhythms of EEG, the EEG data were filtered between 1 Hz and 50 Hz after notch filtering. In the experiment, data from 2 to 7 s were utilized, and baseline correction was performed using data ranging from 0 to 2 s. For the artifact rejection, source derivations based on the center and the neighbor electrodes were calculated, and the boundary electrodes were calculated based on neighbors.

For the DS2, we only retained 26 electrodes, which covered the sensorimotor area. The EEG data were filtered between 1 Hz and 50 Hz after 50 Hz notch filtering. Experimental analysis data were analyzed using 500 samples of data from 2 to 6 s, and baseline correction was performed using data ranging from 0 to 1 s. Then, the 1000 Hz raw EEG signal was downsampled to 125 Hz to reduce the data volume, enhancing the efficiency of signal processing and analysis. In order to validate the effectiveness of the algorithm on data with artifacts, no specific artifact removal processing was conducted. 

For the DS3, the EEG data were filtered between 1 Hz and 50 Hz after 50 Hz notch filtering. In the experiment, data from 2 to 6 s were utilized, and baseline correction was performed using data ranging from 0 to 2 s. Then, the 1000 Hz raw EEG signal was downsampled to 250 Hz, and no specific artifact removal processing was conducted.

For all datasets, the SSA time optimization range was set to within 4 s after the onset of the MI task, enabling unrestricted exploration for the optimal time segment. The frequency optimization range was 1~40 Hz, encompassing the sensorimotor rhythms alpha (8~12 Hz) and beta (14~30 Hz), which are considered to be the main frequency bands related to MI. It can be observed that the three datasets differed in terms of channel count, artifact removal and motor intention. This difference contributed to validating the effectiveness of the algorithm when dealing with data from different channels, with artifacts and data related to different motor imagery tasks.

### 4.2. Whole Framework

**Figure 7:**
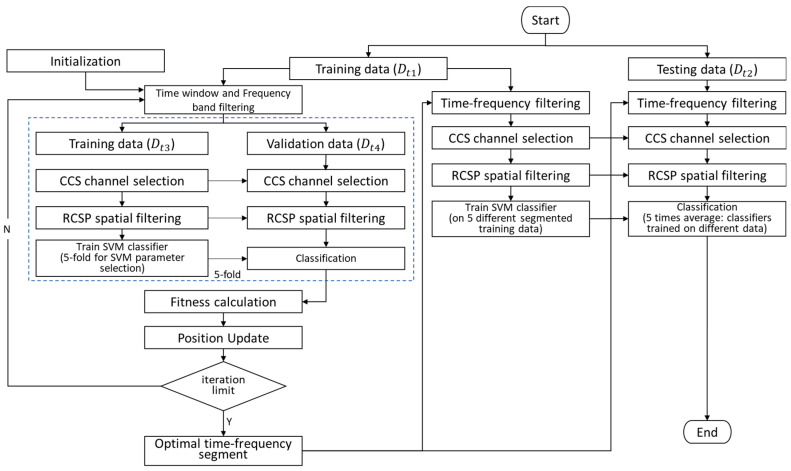
Illustration of the proposed framework.

In this work, we used SSA to determine the optimal time–frequency segments, specifically the time window and frequency band of EEG. Additionally, optimal channels were selected using the CCS method, and the RCSP method was applied to extract features before SVM classification. The framework of the proposed SSA-CCS-RCSP is shown in Figure 7.

The procedure is as follows:

(1) Initialize the positions of n (n = 10) sparrows, corresponding to time–frequency segments;
(2) Split the experimental data into a training set $D_{t1}$ and a test set $D_{t2}$ in a 7:3 ratio;
(3) Apply time–frequency filtering to $D_{t1}$ based on the current time–frequency segment;
(4) Utilize a five-fold cross-validation method, where $D_{t1}$ is split into a training set $D_{t3}$ and a validation set $D_{t4}$ in each iteration (in a 4:1 ratio);
(5) Calculate the optimal channels for $D_{t3}$ using the CCS algorithm and remove irrelevant channel data from both $D_{t3}$ and $D_{t4}$;
(6) Compute RCSP spatial filters on $D_{t3}$ and extract features from both $D_{t3}$ and $D_{t4}$;
(7) Train an SVM classifier on $D_{t3}$ and evaluate the accuracy (fitness) on the test set $D_{t4}$;
(8) Update the time–frequency segments for each sparrow based on the fitness;
(9) Repeat steps (3) to (8) until the predefined iteration limit is reached; in this experiment, the iteration limit was set to 20. The current optimal result obtained is the best time–frequency segment;
(10) Use $D_{t1}$ as the training set and $D_{t2}$ as the test set. The ratios are determined according to the specifications of the datasets providers: 1:1 for DS1, 3:1 for DS2, and 3:7 for DS3. Based on the best time–frequency segment obtained, calculate the accuracy of the current model on the test set according to steps (5) to (7).

In the process of optimizing time–frequency segments based on the SSA, channel selection was accomplished by CCS through the computation of signal similarity across diverse channels. An imperative consideration for effective channel selection is maintaining a high signal-to-noise ratio; consequently, CCS was employed subsequent to time segmentation and frequency filtering. And prior to the classification process, RCSP was employed as a method for feature extraction.

### 4.3. Experimental Results

The performance of the proposed algorithm was evaluated through experimental comparisons using the datasets mentioned earlier. The comparison involved standard feature extraction methods like CSP, RCSP, and their fusion with SSA or CCS. Additionally, FBCSP, which integrates frequency band processing, was included for comprehensive comparison. The classification tasks were executed using an SVM classifier, and the results are presented in Table 1.

The accuracy of the fusion method using SSA or CCS surpassed that of the original algorithm. Moreover, for all datasets, the classification accuracy of our approach was the highest. The results indicate that proposed algorithm effectively enhanced the efficiency of MI decoding.

### 4.4. Analysis of Variance for Results

We conducted a two-way analysis of variance on the experimental results to analyze whether the influence of different algorithms and datasets on classification accuracy was statistically significant. The analysis of variance with two factors was also employed, with the first factor being the algorithm and the second factor being the dataset. The results in Table 2 show that the interaction between the datasets and the algorithms was not statistically significant (*p* > 0.05). However, the algorithm factor had a significant impact on the classification accuracy (*p* < 0.05). The dataset factor significantly influenced the classification accuracy (*p* < 0.05), potentially stemming from individual disparities and variations in collection environments. In Table 3, post hoc analysis with least significant difference shows statistical differences between the SSA-CCS-RCSP and three algorithms implementing a variant of the CSP algorithm alone (namely, FBCSP, CSP, RCSP; *p* < 0.05). This would indicate that adding SSA, CCS, and RCSP to the classification chain will significantly increase the classification performance.

## 5. Discussion

### 5.1. Optimal Time–Frequency Segment Distributions

**Figure 8:**
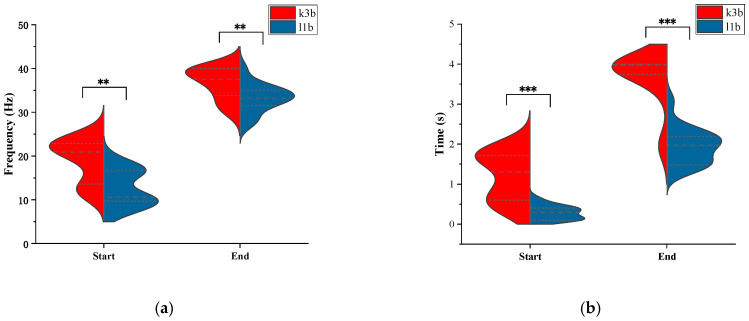
Optimal time–frequency segment distributions. (**a**) Frequency start and end point distribution; (**b**) time segment start and end point distribution. *** and ** above certain lines denote that the distribution of time and frequency between two subjects was significantly different at the 0.001 and 0.01 levels of significance. The two closely spaced dashed lines at the top and bottom represent the maximum and minimum values, while the dashed line in the middle represents the median.

By setting different target channel numbers, the CCS algorithm segmented the EEG signals of subjects K3b and L1b into 10 subsets of DS1 with varying channel numbers. The proposed algorithm was employed to calculate the optimal time–frequency segment. By statistically analyzing the optimal time–frequency bands for different datasets, Figure 8a,b illustrate the distribution of time–frequency bands for two subjects in the DS1.

**Figure 9:**
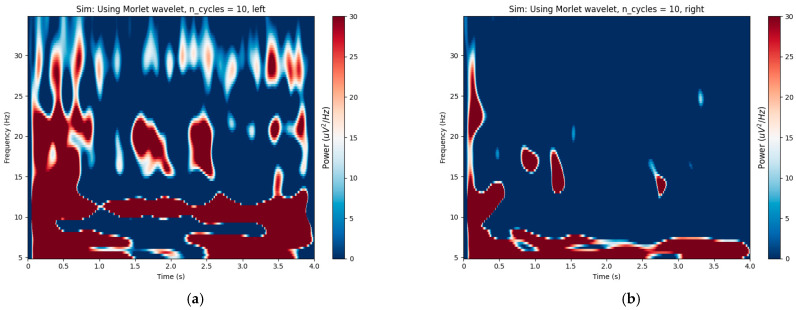

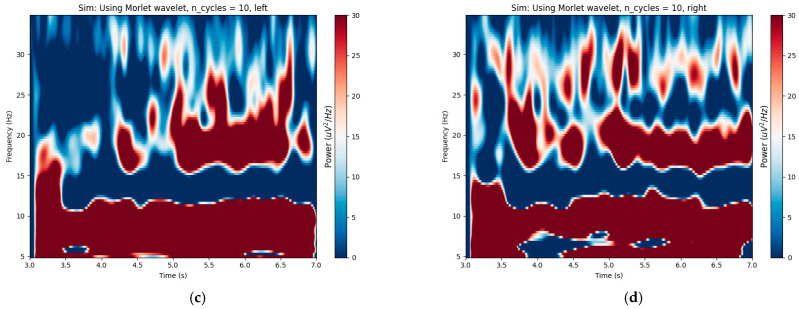
Time–frequency plot with wavelet analysis. (**a**) Left-hand task of participant K3b; (**b**) right-hand task of participant K3b; (**c**) left-hand task of participant L1b; (**d**) right-hand task of participant L1b.

Observing Figure 9a,b, it can be found that around 10 Hz and 20 Hz, there is a significant energy distribution in the signal of subject K3b. At the same time, in Figure 8a, it can be observed that the optimized frequency band for K3b is prominently concentrated around 10 Hz and 20 Hz. The same phenomenon can also be observed in subject L1b from Figure 8b and Figure 9c,d. Additionally, the distribution of time and frequency between two subjects was significantly different, indicating individual differences in EEG signals. From Figure 9, it can be observed that L1B exhibited a significantly lower energy distribution frequency band compared to K3b, and this phenomenon is reflected in Figure 8. It can be inferred that the optimal time–frequency bands obtained by the proposed algorithm effectively reflect the changes in the energy of the EEG signals.

### 5.2. Effect of SSA on Channel Selection

**Figure 10:**
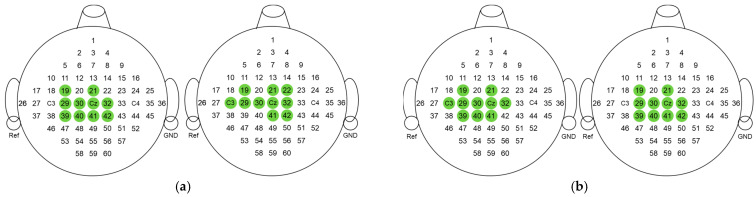
Channel selection results with and without SSA. (**a**) Channel selection results for K3B; (**b**) channel selection results for L1B. The channels selected by CCS are annotated in green.

We investigated the impact of SSA on channel selection in CCS. We applied the SSA algorithm to DS1, and 20 channels were selected by the CCS algorithm for K3B and L1B, as shown in Figure 10. 

The selected channels of the two participants cover areas located in the primary sensorimotor region, which are considered significant areas for ERD/ERS. The overlap between the selected channels and the significant regions of MI-EEG signals suggests that the chosen channels may be neurophysiologically meaningful.

From Figure 10, it can be observed that the channel selection combined with time–frequency optimization yielded results similar to the original CCS. Due to differences in brain activation regions within different frequency bands during the motor imagery task, there were still differences in individual channels. Considering the classification results in Table 1, our proposed method was more effective for different subjects.

### 5.3. Effect of SSA on RCSP Feature Distribution

**Figure 11:**
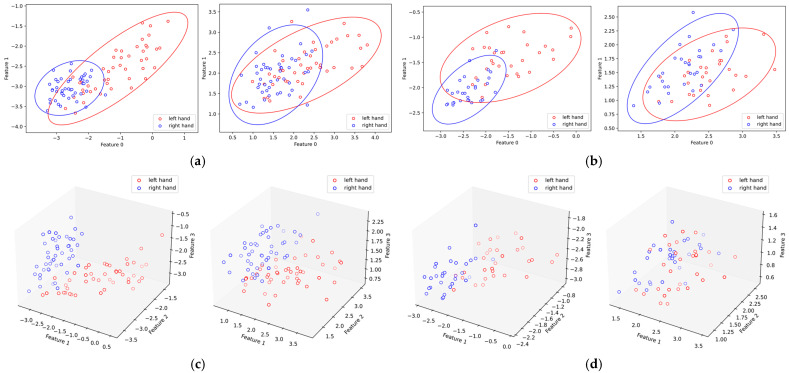
Feature distribution with and without SSA. (**a**) Feature distribution for K3b; (**b**) feature distribution for L1b; (**c**) 3D feature distribution for K3b; (**d**) 3D feature distribution for L1b. The axes represent the feature values extracted by RCSP(m = 2); each dot corresponds to the feature of a trial.

In order to compare the differences in the distributions of features with and without SSA, Figure 11 illustrates the feature distributions of subjects K3B and L1B in DS1. The feature distributions after optimization with SSA became more discriminative, which provides a basis for explaining the improvement in accuracy at the feature distribution level.

### 5.4. Effects of CCS and SSA on Improving Classification

We compared the CSP and RCSP algorithms with their fusion algorithms incorporating CCS or SSA on DS1, DS2, and DS3. The obtained comparison results are presented in Table 4.

Compared with the CSP and RCSP algorithms, the mean accuracy of the CCS fusion algorithm was improved (2.83% and 3.72% for DS1, 2.17% and 2.19% for DS2, 15.58% and 10.25% for DS3, respectively). Compared with the CSP and RCSP algorithms, the mean accuracy of the SSA-RCSP algorithm was improved (3.72% and 5.22% for DS1, 3.92% and 3.94% for DS2, and 13.00% and 7.67% for DS3, respectively). The proposed SSA-CSP-RCSP proposed in this paper achieved the highest accuracy on all datasets, with 99.11%, 87.70%, and 90.50%, respectively.

The results show that the fusion algorithms of SSA and CCS enhanced the classification accuracy by optimizing time–frequency segments and channel selection when compared to algorithms employing non-customized time–frequency segments and utilizing all channels. Furthermore, the fusion algorithm incorporating CCS effectively reduced the number of required channels and decreased the amount of data to be processed.

### 5.5. Performance of SSA with Different Channel Numbers

The channels of the EEG signal datasets are usually not uniform, necessitating an examination of the suitability of SSA in datasets with varying channel numbers. The CCS algorithm segmented the EEG signals into datasets with varying channel numbers, and the SSA-based algorithm was employed to obtain the optimal time–frequency segment.

**Figure 12:**
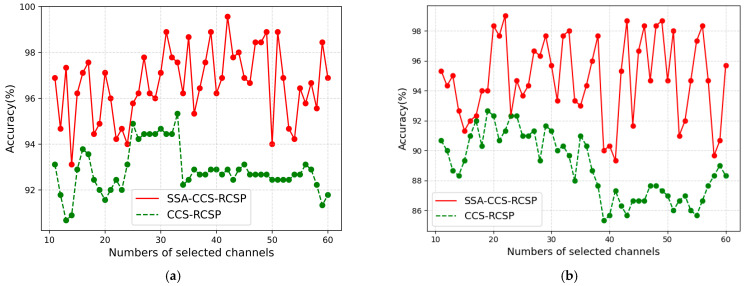
Comparison of classification accuracies between optimized time–frequency segments and non-customized time–frequency segments. (**a**) Classification accuracies for k3b; (**b**) classification accuracies for l1b.

Prior to employing the CCS-RCSP algorithm for extracting signal features, time–frequency filtering was separately performed using the optimized segments and non-customized parameters (0~4 s and 8~30 Hz). The accuracy of classification by SVM is illustrated in Figure 12.

It can be observed from Figure 12 that, with data from different channels, the accuracy of the SSA-CCS-RCSP method consistently exceeded the accuracy of methods based on non-customized time–frequency ranges. The results indicate that this algorithm can be generalized to data with different channel counts.

## 6. Conclusions

To address the problem of poor generalization in algorithms utilizing non-customized time–frequency segments due to the individual variability, this paper proposes a new approach to optimize the time–frequency segment. Further, the CCS was employed to select channels relevant to the MI tasks, while the RCSP was utilized for feature extraction. The performance of the proposed method was evaluated using three datasets: the BCI Competition III Dataset IIIa, the Chinese Academy of Medical Sciences autonomous dataset, and the BCI Competition IV Dataset 1. The results show that the classification efficiency of the proposed algorithm outperformed the algorithms in the comparison group. It effectively addressed the issue of time–frequency segment optimization.

Although this algorithm does consume more computational resources compared to those based on non-customized time–frequency segments, the computational cost is not required for testing (almost real-time), but only for model training (approximately 2000 s is required for data with 59 channels, 1000 sampling points, and 200 trials), and this will not impede its significant importance in practical implementations. Accordingly, we have enough reasons to believe that the proposed methods could significantly improve the MI classification performance. Meanwhile, the proposed methods would potentially contribute to the field of MI-BCI motor rehabilitation systems and assistive devices, which emphasizes the need to customize time–frequency bands for each subject.

In addition, the dataset used in this study was collected from healthy participants, mainly young individuals. Therefore, further work is needed in order to test the proposed algorithm in patients with motor dysfunction.

## Figures and Tables

**Table 1:** Comparison of classification accuracies achieved by CSP, CCS-CSP, RCSP, CCS-RCSP, SSA-RCSP, FBCSP, and SSA-CCS-RCSP, with SVM being the classifier, respectively, on DS1, DS2, and DS3.

Subjects	Accuracy (%)						
	CSP	CCS-CSP	RCSP	CCS-RCSP	SSA-RCSP	FBCSP	SSA-CCS-RCSP
k3b	92.22	94.89	92.22	95.33	98.00	79.38	99.56
l1b	91.33	94.33	88.33	92.67	93.00	53.93	98.67
mean	91.78	94.61	90.28	94.00	95.50	66.66	99.11
A01	73.25	73.25	76.00	79.25	76.25	84.00	87.75
A02	91.50	92.75	91.50	93.00	94.25	95.75	97.25
A03	82.75	86.75	88.75	89.25	89.75	83.50	92.25
A04	88.75	90.25	90.50	91.50	94.75	90.50	96.75
A05	70.00	70.50	67.75	73.25	73.75	72.50	76.75
A06	96.00	96.25	95.00	96.25	97.00	93.75	98.50
A07	59.00	65.50	63.50	65.50	72.50	58.75	72.50
A08	83.50	86.50	80.00	84.50	88.00	90.00	91.00
A09	73.25	73.25	65.25	65.25	67.25	72.75	78.75
mean	79.78	81.67	79.81	81.97	83.72	82.39	87.94
a	63.67	86.67	59.33	91.00	86.00	81.60	94.00
b	72.67	76.67	71.33	76.67	74.33	62.13	82.67
f	75.17	86.00	88.33	89.33	88.00	81.80	91.33
g	75.17	91.33	89.00	92.00	90.33	84.53	94.00
mean	71.67	85.17	77.00	87.25	84.67	77.52	90.50

**Table 2:** Two-way analysis of variance for experimental results.

Subjects	Degree of Freedom	Sum of Squares	Mean Square	F	PR (>F)
dataset	2.0	0.077	0.039	3.517	0.034 *
algorithm	6.0	0.198	0.033	3.002	0.010 *
dataset * algorithm	12.0	0.141	0.012	1.070	0.396
Residual	84.0	0.924	0.011		

* *p* < 0.05

**Table 3:** Post hoc analysis with least significant difference.

Comparison		Mean Difference	Standard Error	t-Value	*p*-Value
CCS-CSP	CCS-RCSP	−0.007	0.038	−0.171	0.865
CCS-CSP	CSP	0.051	0.038	1.328	0.187
CCS-CSP	FBCSP	0.053	0.038	1.387	0.169
CCS-CSP	RCSP	0.039	0.038	1.007	0.317
CCS-CSP	SSA-CCS-RCSP	−0.058	0.038	−1.505	0.136
CCS-CSP	SSA-RCSP	−0.012	0.038	−0.317	0.752
CCS-RCSP	CSP	0.058	0.038	1.499	0.137
CCS-RCSP	FBCSP	0.060	0.038	1.557	0.123
CCS-RCSP	RCSP	0.045	0.038	1.178	0.242
CCS-RCSP	SSA-CCS-RCSP	−0.051	0.038	−1.334	0.185
CCS-RCSP	SSA-RCSP	−0.006	0.038	−0.146	0.884
CSP	FBCSP	0.002	0.038	0.058	0.954
CSP	RCSP	−0.012	0.038	−0.322	0.748
CSP	SSA-CCS-RCSP	−0.109	0.038	−2.833	0.006
CSP	SSA-RCSP	−0.063	0.038	−1.645	0.103
FBCSP	RCSP	−0.015	0.038	−0.380	0.705
FBCSP	SSA-CCS-RCSP	−0.111	0.038	−2.891	0.005
FBCSP	SSA-RCSP	−0.066	0.038	−1.703	0.092
RCSP	SSA-CCS-RCSP	−0.097	0.038	−2.512	0.014
RCSP	SSA-RCSP	−0.051	0.038	−1.323	0.189
SSA-CCS-RCSP	SSA-RCSP	0.046	0.038	1.188	0.238

**Table 4:** Mean classification accuracies achieved by CSP, RCSP, SSA-RCSP, CCS-CSP, CCS-RCSP, and SSA-CCS-RCSP, with SVM being the classifier, respectively, on DS1, DS2, and DS3. The abbreviation NC represents the average number of channels selected across subjects in datasets by CCS under the highest accuracy levels.

Algorithm	Algorithm	DS1		DS2		DS3	
		Accuracy (%)	NC	Accuracy (%)	NC	Accuracy (%)	NC
Spatial Pattern	CSP [4,28,35]	91.78	60	79.78	26	71.67	59
	RCSP [5,7]	90.28	60	79.81	26	77.00	59
SSA Improved	SSA-RCSP	95.50	60	83.72	26	84.67	59
Channel Selection	CCS-CSP	94.61	19	81.67	20.4	85.17	25.25
	CCS-RCSP	94.00	35	81.97	19.7	87.25	35.25
SSA Improved (This study)	SSA-CCS-RCSP	99.11	42.5	87.94	21.4	90.50	25.75

## Data Availability

The DS1 is publicly available online: https://www.bbci.de/competition/iii/ (accessed on 27 December 2023). The DS2 is available with permission from the authors. The DS3 is publicly available online: https://www.bbci.de/competition/iv/ (accessed on 27 December 2023).

## Supplementary Materials

The following supporting information can be downloaded at: https://www.mdpi.com/article/10.3390/s24051678/s1, S1: Patient consent form.
